# Evidence of tuberculosis treatment outcomes among people experiencing homelessness: a scoping review

**DOI:** 10.1186/s12913-025-12230-w

**Published:** 2025-04-02

**Authors:** Claudia Susana Pérez Guerrero, Tiago Augusto Cavalcante Oliveira, Willie Otávio Bueno Bernardi, Stephanie Ribeiro, Jeanne-Marie Stacciarini, Aline Aparecida Monroe, Hugo Fernandes, Paula Hino

**Affiliations:** 1 Federal University of São Paulo, São Paulo, São Paulo Brazil; 2 University of São Paulo, Ribeirão Preto, São Paulo Brazil; 3 University of Michigan, Ann Arbor, Michigan USA

**Keywords:** Tuberculosis, Ill-housed persons, Treatment outcome, Evidence-based practice, Epidemiological monitoring, Public health

## Abstract

**Background:**

Tuberculosis is an infectious, preventable and treatable disease that is socially determined. People experiencing homelessness represent a group that is highly vulnerable to this disease, presenting a challenge for its control and elimination. The aim of this review was to synthesize the existing scientific evidence on the outcomes of tuberculosis treatment in the context of the population experiencing homelessness.

**Methods:**

This scoping review was conducted following JBI guidelines. Six databases were consulted: MEDLINE, Web of Science, Scopus, LILACS, CINAHL and EMBASE. Scientific literature with quantitative or mixed-method approaches may be included, published from 2015 onward, in English, Portuguese and Spanish, involving participants aged 15 years or older. The Rayyan application was used to facilitate the selection process, and a descriptive analysis of the findings was performed.

**Results:**

Fourteen articles were included, comprising primarily cohort studies (*n=* 6) and cross-sectional studies (*n=* 5), along with two ecological studies and a systematic review. Eight articles were from South America (seven from Brazil), three from Europe and three from Asia. The rates of treatment success outcomes ranged from 89.7% to less than 30%, with nine studies reporting rates under 45%. The highest proportion of accumulated unsuccessful treatment outcomes was nearly 70%, with four studies indicating rates between 60% and 66%. Loss to follow-up was the most frequently reported negative outcome (*n=* 9), reaching rates of 53.6%. The “failed” treatment outcome was reported in low proportions, often less than 1% (*n=* 5) and “not evaluated” outcome was reported in half of the studies (*n=* 7). The proportions observed in the systematic review were consistent with these findings. Furthermore, the results revealed significant differences compared with those of the global general population. While both groups exhibited low proportions of treatment failures, other outcomes for the homeless population were markedly poorer.

**Conclusions:**

The homeless population experiences low success rates in tuberculosis treatment, with no study in this review meeting the international treatment success rate target. A comprehensive, collaborative and patient-centered care approach that addresses social determination of health is essential to improve outcomes and enhance health, social care, and educational services tailored to the needs of this population.

**Supplementary Information:**

The online version contains supplementary material available at 10.1186/s12913-025-12230-w.

## Background

Tuberculosis (TB) is an infectious disease that affected more than 10,8 million people in 2023 and is responsible for more than one million deaths worldwide, despite being preventable and treatable and having a long history throughout human civilization. The global incidence rate was 134 new cases per 100,000 inhabitants (95% uncertainty interval= 125–145), reflecting an 8.3% decrease from 2015 [1]. This reduction falls short of the 50% reduction target for 2025 under the End TB Strategy proposed by the World Health Organization (WHO) in 2015. Additional targets of this strategy include achieving at least 90% coverage of notified cases and curing at least 90% of treated cases. Moreover, Sustainable Development Goal (SDG) number three aims to eliminate TB as an epidemic by 2030, underscoring the global importance of addressing this disease [2].

To respond to this problem, access to timely and effective treatment must be guaranteed. Concurrently, clinical follow-up should include a nutritional approach, social protection, and efforts to combat associated stigma [3]. To promote adherence to treatment, it is crucial that care is centered around the needs, values, and preferences of individuals while also upholding their human rights, respect, and dignity [4].

The health-disease process associated with TB is complex and multifaceted. Treatment success is closely related to sociodemographic characteristics, with poverty and social marginalization significantly increasing the risk of infection and hindering treatment adherence. TB is considered a socially determined disease, with evidence indicating that factors such as black or brown race, low educational attainment and living on the street are associated with increased risks of loss to follow-up (LFU) and progression to death [5, 6].

People experiencing homelessness (PEH) are a social group characterized by social inequalities. Compared with the general population, these individuals face more complex needs, a greater prevalence of comorbidities, limited awareness of health needs, and difficulties accessing healthcare services [7]. Owing to factors such as homelessness, inadequate sleep, poor nutrition, substance abuse and ongoing survival struggles, PEH are highly vulnerable to TB and are more susceptible to adverse outcomes of the disease. This situation presents a substantial challenge for TB control and elimination efforts [8–11].

While there are reviews on the occurrence of TB in PEH [11, 12], there is a lack of specific reviews that address the range of TB treatment outcomes within this group. Considering the international guidelines and commitments established to address the disease, this review aimed to synthesize the existing scientific evidence on the outcomes of TB treatment in the context of PEH.

## Methods

This scoping review was conducted in accordance with JBI guidelines [13] and the extension checklist for scoping reviews of the report for systematic reviews and meta-analyses (PRISMA-ScR) [14]. The following steps were followed: defining and aligning the objective, formulating the research question and eligibility criteria, outlining the approach, conducting the database search, selecting evidence, extracting and analyzing the data, presenting and synthesizing the results and drawing conclusions on the basis of the proposed objective [13]. Previously, a review protocol was registered on the Open Science Framework (OSF) website (10.17605/OSF.IO/PFGE5).

The “Population, Concept and Context” (PCC) strategy was employed to formulate the research question [14]. With the population (P) being individuals affected by TB, the concept (C) being the outcome of TB treatment, and the context (C) referring to the experience of homelessness. Thus, the guiding question was as follows: What is the scientific evidence on the outcomes of TB treatment within the context of the homeless population?

TB treatment outcomes were categorized according to the WHO’s definitions [15, 16]:

- Treatment success is the sum of cured and treated patients. Where cured is a pulmonary TB patient with bacteriologically confirmed TB at the beginning of treatment who completed treatment as recommended by the national policy, with evidence of bacteriological response and no evidence of failure. In addition, treatment completed is when a patient completed treatment, as recommended by the national policy, whose outcome does not meet the definition for a cure or treatment failure.
- Death: A patient who died before starting treatment or during the course of treatment.
- LFU: A patient who did not start treatment or whose treatment was interrupted for two consecutive months or more.
- Treatment failed: A patient whose treatment regimen needed to be terminated or permanently changed to a new regimen or treatment strategy.
- Not evaluated: A patient for whom no treatment outcome was assigned.

These last four categories are considered unsuccessful outcomes.

With respect to PEH, there is no universally accepted definition. This definition varies across countries, as it is culturally contextualized on the basis of concepts such as adequate housing and tenure. According to the United Nations (UN) Human Settlements Programme, “Homelessness is a condition where a person or household lacks habitable space with security of tenure, rights and ability to enjoy social relations, including safety”. These include the following categories: people living on streets or other open spaces; temporary or crisis accommodations; severely inadequate and insecure accommodations; and a lack of access to affordable housing [17].

### Search strategy

The search strategy was developed by selecting descriptors from the controlled vocabularies Medical Subject Headings (MeSH) and Health Sciences Descriptors (DeCS) in English, Portuguese, and Spanish. The descriptors and their corresponding synonyms, according to the PCC strategy, are detailed in Supplementary Material 1.

**Fig. 1:**
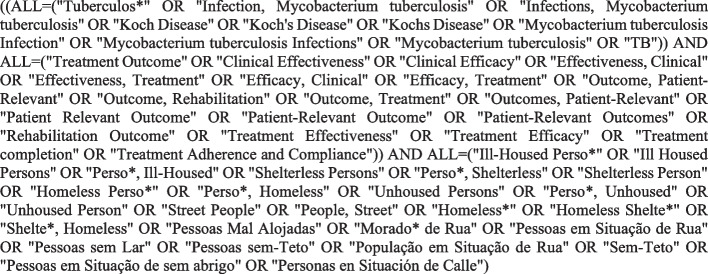
General search strategy

In July 2024, six databases were consulted: Medical Literature Analysis and Retrieval System Online (Medline), Web of Science (WOS), Scopus, Latin American and Caribbean Health Sciences Literature (LILACS), Cumulative Index to Nursing and Allied Health Literature (CINAHL) and Excerpa Medica DataBASE (Embase). Figure 1 shows the general search strategy using the Boolean operators “AND” and “OR” and the truncation symbol “*”. The specific strategy used for each database can be found in Supplementary Material 2.

### Eligibility criteria

The inclusion criteria were primary (original research) and secondary scientific resources (review articles), as well as gray literature (such as dissertations and theses), that employed a quantitative or mixed-methods approach to present the proportion of TB treatment outcomes, and involved participants aged 15 years or older. The search had no geographical restrictions and was conducted in English, Portuguese, and Spanish, with publications from 2015 onward, in view of the start of the End TB Strategy [1]. Exclusively qualitative material, newsletters and government and ministerial documents or sources such as event proceedings, preprints, partial research reports, editorials and letters to the editor were excluded, as were studies that dealt with drug-resistant TB, latent TB infection (LTBI), meningoencephalitic and osteoarticular TB, or in children under 14 years old, since in these situations, the treatment regimens are special [9].

### Screening and data extraction

The materials found in each database were exported to the Rayyan application developed by the Qatar Computing Research Institute [18], where duplicates were removed. The remaining materials were then assessed independently by two researchers (CSPG and TACO) according to the eligibility criteria. Initially, titles and abstracts were reviewed, followed by a full-text examination of the articles. Any disagreements were resolved through discussion with a third author (SR). Additionally, a manual search was conducted by examining the bibliographical references of the selected studies and searching for gray literature. An adapted data extraction form [13] was employed by two investigators (CSPG and TACO). The evidence was analyzed descriptively, with a focus on the proportions of TB treatment outcomes in PEH. International research ethics regulations were followed. Since the study did not involve direct contact with human participants, it did not require approval from an ethics committee.

## Results

**Fig. 2:**
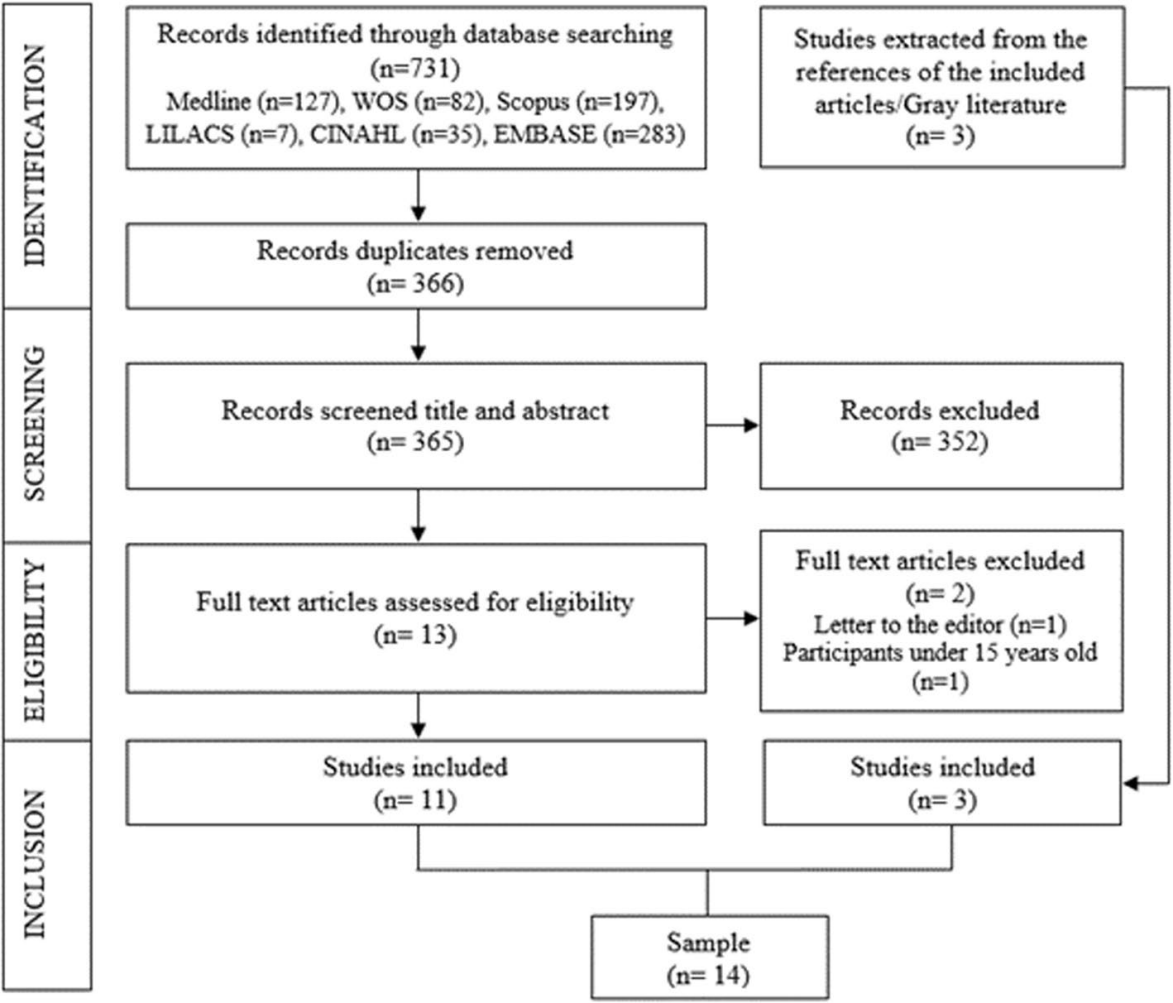
Flowchart of article selection

The initial database search yielded 731 documents. After applying exclusions and conducting a manual search, 14 scientific articles were included (Fig. 2).

Table 1 provides a summary of the main characteristics of the articles [10, 11, 19–30]. The articles showed heterogeneity in terms of the study population, approach and proportion of TB treatment outcomes. In the section “Proportions of TB treatment outcome in PEH”, the outcomes were categorized as “success” and “unsuccessful”. Most studies group “cure” and “treatment completed” under the first category; however, some reported only “cure” [10, 21] or “treatment completed” [22]. In the “unsuccessful” category, each type of treatment outcome is detailed when reported.

**Table 1:** Summary of the main characteristics of the studies reviewed

**Author, country, year**	**Title**	**Study type, data source**	**Objective**	**Participants**^**a**^	**Proportions of TB treatment outcome in PEH**^**b**^	
					**Success**	**Unsuccessful**
Pavinati et al., Brazil, 2023 [10]	Geoprogrammatic disparities in the performance of tuberculosis indicators in the homeless population in Brazil: an ecological approach	Ecological, *Sistema de Informação de Agravos de Notificação* (SINAN)	To analyze the performance and spatial distribution of TB control indicators in PEH in Brazil.	New TB cases, between 2015–2021, in PEH aged 20 to 59 years. *N=* 8.402	38% (*n=* 3.193)	Death: 14% (*n=* 1.176) LFU: 36% (*n=* 3.025)
Rodrigues et al., Brazil, 2023 [19].	Factors associated with unsuccessful tuberculosis treatment among homeless persons in Brazil: A retrospective cohort study from 2015 to 2020.	Retrospective cohort, SINAN	To evaluate the factors associated with unsuccessful TB outcomes among PEH compared to those with shelter.	New TB cases, between 2015–2020, with individuals between 18 and 90 years old, separated into PEH and the general population. *N=* 7.749	39.84% (*n=* 3.086)	Death: 14.37% (*n=* 1.114) LFU: 36.02% (*n=* 2.791) Failed: 0.06% (*n=*5) Not evaluated: 9.71% (*n=* 753)
Gabdullina et al., Kazakhstan, 2023 [20].	COVID-19 pandemic and other factors associated with unfavorable tuberculosis treatment outcomes-Almaty, Kazakhstan, 2018–2021.	Retrospective cohort, National electronic database of Kazakhstan	To evaluate the association of the COVID-19 pandemic period and risk factors related to adverse TB treatment outcomes among people newly diagnosed with TB in Almaty, Kazakhstan, from 2018 through to 2021.	New TB cases between 2018–2021 aged at least 18 years, separated into different risk groups. *N=* 18	55.55% (*n=* 10)	44.44% (*n=* 8)
Scholze et al., Brazil, 2022 [21].	Tuberculosis among People Living on the Street and Using Alcohol, Tobacco, and Illegal Drugs: Analysis of Territories in Extreme Vulnerability and Trends in Southern Brazil.	Ecological, SINAN	To analyze territories where there is a concentration of people diagnosed with TB, who are homeless and who chronically use alcohol, tobacco and illicit drugs, and to analyze trends in this health condition in southern Brazil.	TB cases in homeless people between 2014–2018 aged 18 or older in the state of Paraná, Brazil, who use alcohol, tobacco or illicit drugs. *N=* 560	35.8% (*n=* 200)	Death: 16.3% (*n=* 91) LFU: 27% (*n=* 151) Failed: 2.3% (*n=* 13) Not evaluated: 16.3% (*n=* 91)
Crosby et al., England, 2022 [22].	Outcomes of a residential respite service for homeless people with tuberculosis in London, United Kingdom: a cross-sectional study.	Cross-sectional, London TB Register	To compare the characteristics and treatment outcomes of patients treated in the Residencial Respite Service (RRS) with patients treated in standard care and to estimate the association between treatment in the RRS and treatment outcomes.	TB cases between 2010–2019 aged 18 or older, separated into treated in RRS and treated in the usual way. *N=* 78 (treated in RRS)	89.74% (*n=* 70)	Failed: 10.25% (*n=* 8) (95% CI = 5%–20%).
Santos et al., Brazil, 2021 [23].	Analysis and comparison of tuberculosis treatment outcomes in the homeless population and in the general population of Brazil.	Cross-sectional, SINAN	To compare the rates of treatment success, LFU and death from TB between the PEH and the general population in Brazil and its regions in 2018.	New TB cases in 2018, separated into PEH and the general population. *N=* 1.530	39% (*n=* 597, 95% CI= 36.5–41.4)	Death: 8,1% (*n=* 124, 95% CI= 6.8–9.6) LFU: 28.8% (*n=* 441, 95% CI= 26.6–31.1)
Macedo et al., Brazil, 2021[24].	Vulnerable populations and the outcome of tuberculosis cases in Brazil.	Cross-sectional, SINAN	To evaluate the association between the population deprived of liberty (PDL) or PEH and the failure of TB cases diagnosed in Brazil in 2015.	TB cases in 2015 in individuals over 15 years old, in the general population, PDL and PEH *N=* 2.782	34.7% (*n=* 965)	Death: 11.6% (*n=*323) LFU: 37.7% (*n=* 1.048) Failed: 1.7% (*n=* 48) Not evaluated: 14.3% (*n=* 398)
Hino et al., Brazil, 2021 [11].	Tuberculosis in the street population: a systematic review.	Systematic review, PubMed, EMBASE, LILACS and Scientific Electronic Library Online (SciELO)	To analyze the evidence available in the literature on the occurrence of TB in PEH.	Seven papers reviewed in 2018.	Cure: 35.2%; 44.1%; 55%; 77%, Treatment complete: 85%	LFU: 2.7%; 12%; 21.8%; 24.8%; 39.0% Death: 7.2%; 8%; 10.5%; 11.1%; 19.7%, Not evaluated: 21.9%; 23.5%; 31%. Failed: 0.4%; 1.4%; 1.6%
Kozhoyarova et al., Kyrgyzstan, 2020 [25].	Who is doing worse? Retrospective cross-sectional study of TB key population treatment outcomes in Kyrgyzstan (2015–2017).	Cross-sectional, National TB Registry of the National TB Control Center of Kyrgyzstan	To investigate risk factors and treatment outcomes among different key TB populations (internal migrants, PEH, injecting drug users and ex-prisoners) in the period 2015–2017 in the Chuy region (which includes its capital Bishkek) in Kyrgyzstan.	TB cases between 2015–2017, with individuals over 18 years old, separated into different risk groups *N=* 237	29.1% (*n=* 69)	Death: 21.9% (*n=*52) LFU: 45.1% (*n=* 107) Failed: 3.8% (*n=*9)
Kim et al., South Korea, 2019 [26].	Impact of Housing Provision Package on Treatment Outcome Among Homeless Tuberculosis Patients in South Korea.	Prospective cohort, Three Seoul hospitals and the National Notification Data	To evaluate the effect of a housing provision package on the outcomes of PEH TB treatment in Seoul.	New or previously treated TB cases in PEH between 2016–2018, over 49 years old, separated by type of treatment *N=* 318	80.8% (*n=* 257)	Death: 6.6% (*n=* 21) LFU: 3.7% (*n=*12) Failed: 8.8% (*n=*28)
Gómez et al., Colombia, 2019 [27].	Homelessness and HIV: A Combination Predictive of Poor Tuberculosis Treatment Outcomes and in Need of Innovative Strategies to Improve Treatment Completion.	Retrospective cohort, Antioquia Regional Secretariat for Health and Social Protection	To determine the risk factors associated with unsuccessful TB treatment in Antioquia’s human immunodeficiency virus (HIV)-seropositive and PEH, compared with PEH without HIV, and compared with non–HIV-infected and non-PEH with TB.	Susceptible TB cases between 2014–2016 in HIV-positive people and/or PEH. *N=* 544	34.1% (*n=*186)	Death: 4.4% (*n=* 24) LFU: 53.6% (*n=* 292) Failed: 0.7% (*n=* 4) Not evaluated: 6.9% (*n=* 38)
Ranzani et al., Brazil, 2019 [28].	The impact of being homeless on the unsuccessful outcome of treatment of pulmonary TB in São Paulo State, Brazil.	Cohort, TBWEB	To determine the association between homelessness and the unsuccessful outcome of treatment of newly diagnosed with pulmonary TB patients in São Paulo, Brazil, from 2009 to 2013.	New cases of pulmonary TB between 2009–2013, aged 15 or older, separated into PEH and general population. *N=* 1.726	42.7% (*n=* 737)	Death: 10.5% (*n=* 181) LFU: 39.0% (*n=* 674) Failed: 0.4% (*n=* 7) Not evaluated: 7.4% (*n=* 127)
Dias et al., Portugal, 2017 [29].	Tuberculosis among the homeless: should we change the strategy?	Retrospective cohort, National census and National TB Surveillance System	To evaluate the incidence rate of TB and the treatment outcomes among PEH in Portugal and to identify predictors of treatment failure.	All TB cases between 2008–2014 separated into PEH and the general population. *N=* 734	60.4% (*n=* 443)	Death: 14.4% LFU: 9.4% Failed: 0% Not evaluated: 15.7%.
Korzeniewska-Koseła et al., Poland, 2015 [30].	Tuberculosis in homeless persons in Poland.	Cross-sectional, Nacional TB Register of Poland	To compare the characteristics of TB in PEH and other patients.	TB cases registered between 2004–2013, separated into PEH and general population. *N=* 2.104	44.1% (*n=* 927)	Death: 7.2% (*n=* 152) LFU: 24.8% (*n=* 522) Failed: 0.4% (*n=* 8) Not evaluated: 23.5% (*n=* 495)

^a^N refers only to PEH

^b^CI when reported

Most of the studies were published between 2020 and 2024 (*n=* 9) and in English (*n=* 10), followed by Portuguese (*n=* 4). In terms of geographical distribution, the majority of the articles covered the Brazilian scenario (*n=* 7) out of a total of eight publications from South America, three from Europe and three from Asia. Specifically, nine articles were produced across three countries with high TB burdens: Brazil, Korea and Kazakhstan. In terms of study design, the cohort method was used most frequently (*n=* 6), followed by a cross-sectional design (*n=* 5), two ecological studies and a systematic review. All the studies used local TB electronic surveillance databases as a data source, with the exception of the review.

With respect to data analysis, all the studies used descriptive statistics [10, 11, 19–30], chi-square tests or Fisher’s exact tests were used mainly to measure relationships between variables [19, 20, 23, 24, 26, 28–30], and different logistic regression models were applied to assess the associations between different variables and the outcome of treatment [20–22, 24–26, 28–30]. With respect to the statistical programs employed, various versions of STATA software [21, 23, 25–28], R software [21, 22, 24, 29], SPSS [21, 27, 30], QGIS [10], and Excel [27] stand out.

Notably, eight studies reported receiving approval from an ethics committee [10, 19–21, 24–28], only one of which had direct contact with the participants [26]. Others stated that there was no need [22, 23, 29].

### Evidence of TB treatment outcomes in PEH

**Fig. 3:**
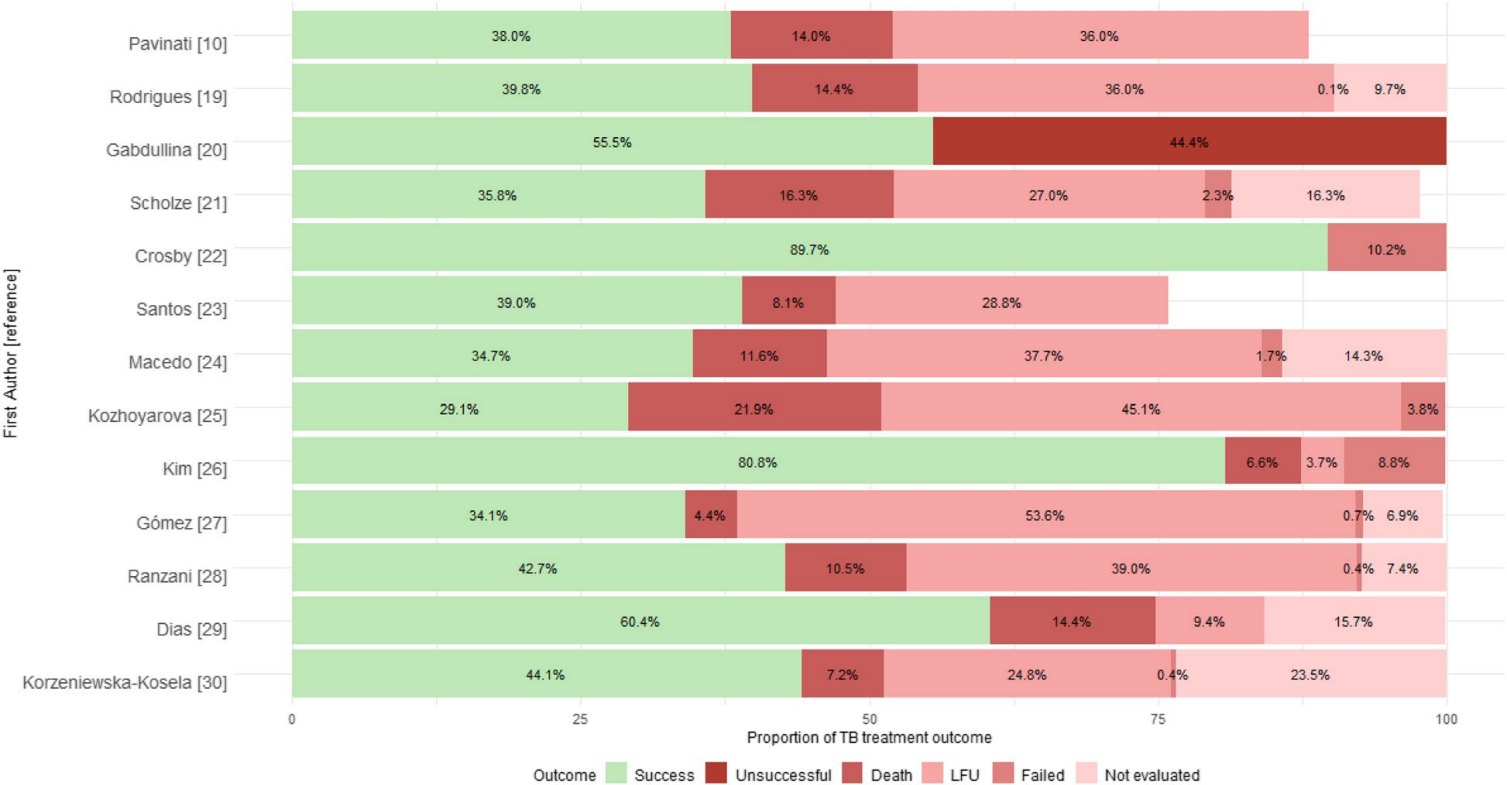
Proportion of tuberculosis treatment outcomes in people experiencing homelessness, by study. (The results of the systematic review [11] were not included.)

The proportions of TB treatment outcomes in PEH are heterogeneous (Fig. 3). In terms of the success rate, an English study comparing patients treated with a residential service to those receiving standard treatment reported the highest success rate among the evaluated studies, reaching 89,7% (*n=* 70/78) [22]. This was followed by another study that reported a success rate of 80% (*n=* 257/318), which assessed the impact of a housing provision package on treatment outcomes [26]. With lower proportions, two studies reported similar success rates: 56.5% (*n=* 10/18) [20] and 60.4% (*n=* 443/734) [29]. In contrast, more than half of the studies (*n=* 9) reported success rates of less than 45% [10, 19, 21, 23–25, 27, 28, 30]. The lowest success rate did not reach 30% (29,1%, *n=* 69/237) [25].

With respect to unfavorable outcomes, half of the studies reported all four types of unfavorable outcomes defined by the WHO [19, 21, 24, 27–30]. Other studies reported only death, LFU, and failed outcomes [25, 26], whereas some focused solely on death and LFU [10, 23], only failed outcomes [22], or simply reported unsuccessful outcomes without further specification [20].

The highest cumulative proportion of TB cases without successful treatment was reported to be 70.8% (*n=* 168/237) in a study conducted in Kyrgyzstan [25]. Four Latin American studies reported rates of 65.8% (*n=* 358/544) [27], 65.3% (*n=* 1.817/2.782) [24], 61.7% (*n=* 346/560) [21] and 60.2% (*n=* 4.663/7.749) [19].

The most frequently reported unfavorable outcome was “LFU”, identified as the most significant negative result in nine out of 13 articles. Notably, it surpassed successful outcomes in three studies [24, 25, 27]. The study that reported the highest rate of this category of treatment outcome was conducted in Colombia, which compared risk factors with variables such as PEH and HIV, showing an LFU rate of 53.6% (*n=* 292/544) [27].

The next most prevalent negative outcome was “death”, with the highest observed rate of 21.9% (*n=* 52/237) [25], followed by 16.3% (*n=* 91/560) [21]. Additionally, three studies reported that approximately 14% of people died with or due to TB: 14.4% (*n=* 1.114/7.749) [19], 14.4% (*n=* 106/734) [29] and 14% (*n=* 1.176/8.402) [10]. Another unfavorable outcome that was reported in fewer studies was “not evaluated” (*n=* 7), with reported rates ranging from 6.9% (*n=* 38/544) [27] to 23.5% (*n=* 495/2.104) [30].

The final outcome, labeled ‘failed’, had the lowest proportion among the results. In five articles, it accounted for less than 1% of the sample [19, 27–30]. However, the highest rates were reported in studies that achieved the greatest success rates: 10.25% (*n=* 8/78, 95% CI= 5%–20%) [22] and 8.8% (*n=* 28/318) [26]. Moreover, not all the studies evaluated included the full set of participants when presenting the different treatment outcomes, as some studies reported fewer participants in the overall sample [10, 21, 23].

The systematic review [11] included seven studies that explored the occurrence and epidemiological profile of TB in the PEH. These studies employed various methodologies and objectives, resulting in a broad spectrum of treatment outcome rates, which is why they were not incorporated into the previously mentioned results. Nonetheless, the profile observed in the systematic review was consistent with those reported in the current review. Success rates reported as a cure ranged from 35.2% to 77%, with one study reporting an 85% treatment completion rate.

In terms of unsuccessful outcomes in the studies in the systematic review [11], the most prevalent was LFU, with rates ranging from 2,7% to 39%. Dead was the next most common outcome, occurring in proportions between 7.2% and 19.7%, both LFU and death reported by five of the seven surveys reviewed. Notably, none of the studies indicated that unfavorable outcomes exceeded favorable outcomes. The “not evaluated” outcome was reported in three studies, with rates ranging from 21.9% to 31%. The ‘failed’ outcome had the lowest representation, approaching 1% of the sample. Importantly, two of the articles analyzed by the systematic review were also included in the present scoping review [28, 30].

Studies have investigated the outcomes of TB treatment among PEH patients via various approaches. Notably, research comparing TB cases between PEH and the general population (or non-PEH) consistently revealed less favorable treatment outcomes among PEH [19, 23, 28–30]. When PEH were compared with the general population, they were found to be twice as likely to experience unsuccessful treatment (adjusted hazard ratio [HR]= 2.04, 95% incidence coefficient [CI]= 1.82–2.28) [19] or to have a fivefold higher risk (adjusted odds ratio [OR]= 4.96, 95% CI= 4.27–5.76) [28]. Additionally, PEH has a higher mortality rate (14.4%) than does the general population (5.4%) [29].

Another approach involves characterizing only PEH, examining their geographical distribution, other risk conditions and the provision of housing as part of their care [10, 11, 21, 22, 26]. When treatment outcomes were compared by type of care received, it was observed that individuals treated in long-term care institutions for PEH with TB had higher odds of treatment success than those receiving standard care. Patients treated with residential services were nearly three times more likely to complete treatment (OR 2.97, 95% CI= 1.44–6.96) [22] and up to 17 times more likely to achieve a successful treatment outcome (adjusted OR= 17.02, 95% CI= 6.76–42.81) [26].

Studies comparing PEH and other population groups have also been identified [20, 24, 25, 27, 29]. PEH had a greater probability of treatment failure (adjusted risk ratio [RR]= 2.94, 95% CI= 1.80–4.80) than did alcohol consumption (adjusted RR= 2.58, 95% CI= 1.83, 3.62) and living with HIV (adjusted RR= 2.72, 95% CI= [1.99, 3.72) [20]. Additionally, individuals with TB-HIV coinfection were 2.1 times more likely to experience unsuccessful treatment than non-HIV-infected patients were (OR, 95% CI= 2.135, 1.312–3.494) [29]. Another study revealed that the TB-HIV coinfected homeless group had a greater risk of adverse outcomes than the non-TB-HIV coinfected and non-homeless groups did (adjusted RR, 95% CI= 1.65, 1.39–2.38) [27].

In addition, compared with the PDL group, being homeless was identified as a risk factor for treatment failure (adjusted OR= 2.38, 95% CI= 2.17–2.61) [24]. Furthermore, compared with the migrant group in another article, the risk of an unsatisfactory outcome was 11 times greater (OR= 11.2; 95% CI= 7.4–17.0) [25].

With respect to the secondary outcomes of interest, the authors collected other PEH variables from the databases, such as sociodemographic variables (age, gender, color/race and education level, and to a lesser extent, financial support or health insurance); clinical factors (including alcohol, tobacco and drug use, HIV/Acquired immunodeficiency syndrome (AIDS) diagnosis and mental health conditions); and TB case-related factors (type of entry, clinical form and use of directly observed therapy [DOT], with the place of diagnosis and antibiotic resistance being reported to a lesser extent).

In PEH, TB predominantly affects young males [11, 19–30], with a black or brown skin color [11, 19, 23, 24, 28] and a low degree of education [19, 23, 28]. A late diagnosis of TB has been noted among PEH [27, 28, 30], and the disease predominantly and almost exclusively presents in the pulmonary form [11, 19, 22–25, 30]. Failure or difficulty in implementing DOT has been identified as a critical factor in disease management and is associated with increased mortality and other unfavorable outcomes [10, 11, 19, 27]. In the absence of DOT, there are more than 18.3 times fewer chances of successfully completing treatment (adjusted HR 18.37, 95% CI= 12.23–27.58) and 15.6 times greater chances of death (adjusted HR 15.67, 95% CI= 4.79–51.15) [19]. Additionally, the studies primarily assessed new TB cases, as opposed to the high readmission rate after LFU in this population group [24, 30].

Although no formal quality assessment tool was employed, studies were evaluated on the basis of the structure of scientific writing, researchers’ affiliations, and journal data to mitigate the risk of bias from selecting low-quality studies. Therefore, some inconsistencies were observed in the data reported in the bodies of the manuscripts and in the tables, figures or graphs [19, 22–24, 26–28]. Issues were also noted in the assessment of the outcome [24, 26], the lack of data on some variables [21] and the omission of some databases used [30]. As limitations, the studies highlighted the lack of measurement or absence of data on several important variables, such as substance use, nutritional status, viral load, mental illness or residence use [19, 20, 22–24, 26, 27].

## Discussion

The two studies that reported the highest levels of success in this review were conducted in high-income countries and took a unique approach by assessing patients who received comprehensive care. This included not only pharmacological treatment but also social and health support, such as housing, nutrition, and assistance from a multidisciplinary team [22, 26]. Despite their successes, these studies still fell short of the WHO’s recommended target of at least 90%, indicating that the vast majority of studies (*n=* 12) were well below international criteria for TB control [1].

Globally, in 2021, treatment outcomes for TB in the general population revealed an 88% success rate. The failure rate was 0.72%, deaths accounted for 4%, 3.5% of patients were LFU, and 4.2% were not evaluated [15]. In contrast, the results of this review highlight significant differences compared with those of the PEH, which shares only the similarity of a low proportion of treatment failures.

The studies reporting a nearly 60% success rate were retrospective cohort studies from upper-middle- and high-income countries [20, 29], whereas most studies reporting success rates of less than 50% were from upper-middle-income countries [10, 19, 21, 23, 24, 27, 28]. Notably, the highest proportion of treatment failure was reported in the study conducted in the only low-income country among the selected studies [25]. It has been demonstrated that economic issues are a determining factor in the control of TB. PEH has an initial probability of a cure close to zero without investment, and this group requires more investment to achieve the expected probability of a cure than other priority groups do (people who use drugs, living with HIV, pregnant women and immigrants) [31].

Compared with the general population, PEH has a greater risk of unfavorable outcomes. The high mortality burden may be attributed to health issues related to poverty, comorbidities, or inadequate access to healthcare [32]. The findings of this review are in line with those of other studies, which reported that PEH was associated with a 3.2-fold increase in the odds of unsuccessful treatment outcomes (OR= 3.23) [33] and a 2.2-fold increased likelihood of mortality during treatment (OR= 2.26) [32]. In this context, it is crucial to recognize that an individual’s living and working conditions, shaped by their social class and immersed in the current capitalist system, play a fundamental role in determining their health outcomes [34].

The lack of housing not only limits access to healthcare services [35] and also increases the risk of illness, as individuals prioritize immediate survival needs, such as safety and food, over health [36]. This phenomenon is part of harmful structural processes that are accelerating globally and are intrinsically linked to the exponential growth of inequity [37]. In this context, the concept of vulnerability should be understood as the identification of weaknesses and the capacity to address health issues. At its core, vulnerability reflects the ability of individuals and social groups to face, withstand, and recover from these challenges. As an indicator of inequity and social inequality, vulnerability must be approached in a multidimensional way, encompassing individual, programmatic, and social perspectives [38].

The sociodemographic profile of TB among PEH is characterized by a predominance of young men of economically active age with black or mixed races. In 2023, adult men accounted for the highest global burden of TB, comprising 55% of the estimated total cases [1]. Men are often more mobile and may experience higher rates of TB due to greater tendencies to engage in smoking, alcohol consumption, and drug use, and in general, they experience longer delays than women in accessing TB care [39]. A study evaluating seven high-income countries reported that TB incidence rates were higher among men in almost all age groups than among women [40]. This further highlights the significant influence of gendered cultural norms on TB outcomes; thus, a gender-sensitive approach is crucial for TB control, focusing on how gender is constructed, performed, and challenged during TB diagnosis and treatment rather than merely emphasizing epidemiological differences based on sex [41]. Moreover, the lack of data on homelessness among women complicates the understanding of gender-specific differences and complexities in TB outcomes [40].

In Brazil, the country of origin for half of the studies reviewed, a similar demographic profile is observed among PEH, the population with TB, and PEH with TB. Most of these individuals are male, aged between 30 and 49 years, and identify as black or mixed race [42–44]. When black and mixed-race populations constitute 55.5% of the country’s demographic data [45], black individuals are associated with poorer TB treatment outcomes than white individuals are, as evidenced by an adjusted OR of 1.20 (95% CI= 1.14–1.28) [24] and an adjusted OR of 1.16 (95% CI= 1.06–1.28) [28]. In a state of the United States, non-Hispanic black individuals had a TB incidence rate that was 6.21 times higher (95% CI= 4.83–7.99) than that of non-Hispanic whites [46]. These findings underscore the existence of racial inequalities in TB incidence rates.

The studies alluded to the existence of the End TB Strategy and its challenges [10, 20–22, 26, 27]. This highlights the need for urgent actions that could impact the TB landscape among PEH. Key recommendations include investing in specific public policies and targeted services [10, 11, 19, 21, 27, 28]; encouraging research to help develop new prevention, diagnosis and treatment strategies; and implementing social protection networks, which are essential for promoting health equity and reducing poverty [10, 11, 19, 21, 22, 26, 28].

Health services that are responsive to PEH, staffed by professionals trained in the needs and approaches of this group are required to improve care for PEH when they are affected by TB [10, 20–23, 27]. However, stigma and dehumanization remain significant barriers to accessing healthcare, diagnosing, and adhering to TB treatment among populations such as the PEH [36, 47]. A study revealed that, from the perspective of health professionals, discrimination against the PEH and the provision of inferior treatment are widespread; in turn, this study also highlights that, from the individual’s perspective, fear of mistreatment and the desire to protect their dignity contribute to their avoidance of healthcare facilities [35]. Ultimately, access to services is only valuable if these services meet sufficient quality standards to ensure effectiveness [39].

PEH often receive diagnoses at advanced stages of disease, abandon treatment, and exhibit low medication adherence, alongside limited use of primary, preventive, and outpatient care services [48]. This group frequently refrains from seeking health services [29] because of factors that encompass individual, social, and structural aspects, which can impact the achievement of operational TB treatment coverage indicators. Negative experiences in seeking healthcare can significantly affect individuals’ behavior and interactions with healthcare services, such as feelings of contested worthiness, rejection, loss of confidence, unwelcome treatment, and hesitancy from healthcare providers to engage meaningfully [49]. These negative experiences have contributed to a reluctance to engage in healthcare services in the future. On the other hand, positive experiences have encouraged health-seeking behavior and service engagement, upheld dignity, and decreased feelings of shame among PEH [50]. Additionally, relationships of trust between people with TB and health teams impact adherence to treatment [51].

Research has shown that social support, including addressing needs such as access to healthcare, housing, and food, is essential for achieving better treatment outcomes for TB in street contexts [22, 26]. Furthermore, income transfer programs or incentives are also recommended [19, 21, 24, 27, 28, 30]. This aligns with some of the Stop TB Partnership strategies, relating the need to strengthen collaboration among health and social programmes, with activities such as poverty alleviation, policies, cash transfers, nutritional support programmes, social security benefits, urban housing initiatives, and compensation schemes [39].

Social support and mental health care with the provision of housing for PEH contribute to adherence to TB treatment [51]. “Social protection is the set of policies designed to reduce and prevent poverty and vulnerability throughout the life cycle, which contributes to preventing homelessness” [17]. Evidence shows that social protection measures for individuals affected by TB, including rights to nutrition, income, housing, and social assistance and security, improve nutritional status and quality of life. These measures reduce catastrophic costs, increase access to healthcare, and promote treatment adherence, resulting in better treatment outcomes [52]. The right to housing, encompassed in SDG numbers one and 11, “is more about understanding the house as a place to dwell, as it corresponds to the ability of people to live free from harm and discrimination” [17]. Although international organizations recommend social protection to prevent homelessness, there is no pronouncement on how to address the causes and consequences of underlying structural inequalities [53].

Studies have confirmed that actively searching for symptomatic respiratory conditions in PEH is an effective strategy [11, 27, 28, 30]. Early diagnosis and effective treatment are fundamental for controlling TB. This approach can be facilitated by the presence of health and social care teams in territories with greater proximity to PEH, such as the *Equipe de Consultório na Rua* in Brazil [11, 19, 21, 23, 24] or Antioquia’s homeless population system in Colombia [27], which has been shown to facilitate improvements in TB prevention and diagnosis, as well as in maintaining treatment until successful outcomes are achieved. Thus, strong collaborations that integrate existing social services with TB care can enhance adherence in populations such as PEH [10, 11, 23, 33, 38].

Another recommendation emphasized the need for public policies aimed at reducing the consumption of psychoactive substances [21]. This is a multifactorial problem, so actions should be guided and formulated on the basis of intersectoral public health interests rather than being limited to individual interventions [54]. This is particularly important given that PEH who use alcohol [11, 21, 24, 28] and other illicit drugs [11, 28, 29] are related to worse TB treatment outcomes. However, one study in this review revealed that PEHs who consume alcohol are less likely to fail TB treatment, possibly because their unique health service access may enhance treatment outcomes [19]. The use of substances such as alcohol or drugs was a predictor of treatment failure (OR 4.0, CI 95%= 1.06–15.2), with homelessness being an even stronger predictor (OR 6.7, CI 95%=1.2–36.3). A collaborative approach between TB and substance-use services could provide a comprehensive solution to these complex challenges [55].

Another aspect to consider is TB-HIV coinfection, since HIV is one of the main clinical risk factors for TB, which is the leading cause of death among people living with HIV [2]. The evidence indicates a high rate of coinfection (17.3% [28], 20.38% [19], 20,4% [10] and 22% [11]) and a risk factor directly associated with TB treatment failure (adjusted RR 1.65, CI 95%= 1.39–2.38) [27], (OR 2.135, CI 95%= 1.312–3.494) [29]. Conversely, a low proportion of HIV testing has been detected in PEH with TB (76,9%) [10], coupled with significant gaps in this variable in the databases, from 25.4% (*n=* 438) [28] to 50,3% (*n=* 274) [27]. When the WHO recommends that all TB cases be tested for HIV [2], this represents another target that remains unmet within this social group.

To reduce the accentuated presence of TB-HIV coinfection among PEH, integration between TB and HIV control programs is needed [10]. One strategy involves co-locating HIV and TB testing and treatment services, which leads to improved early outcomes for TB-HIV comorbidity, including increased rates of HIV testing, initiation of antiretroviral therapy, and enhanced detection of TB cases [56]. In PEH with TB, point-of-care testing for HIV, in which finger-prick blood or saliva samples are analyzed, may be useful because it provides preliminary results at the same patient encounter where testing is performed. Pre- and post-test counseling should be provided, linked to HIV care, and the mandatory steps should be continued [57]. Moreover, improving management across different TB patient profiles, such as individuals presenting with extrapulmonary TB, is possible [58].

Studies have indicated that in compulsory notification databases, there is a large amount of missing data, and self-declaration of homelessness can lead to underreporting of this information [10, 11, 19, 22–24, 28, 29]. However, only one study noted that the registration forms were properly completed [25]. This evidence of marginalization, together with the lack of international quantification of PEH, hinders intervention strategies for TB control [8].

Notably, the proportion of PEH has increased considerably, especially since the coronavirus disease 2019 (COVID-19) pandemic, which has intensified income inequalities between countries [59] and necessitated the restructuring of TB programs, resulting in limitations in human and material resources [60, 61]. COVID-19 has affected follow-up and adherence to TB treatment [62], impacted TB diagnosis and notifications, and been associated with high lethality rates [20] compared with the prepandemic period [59].

In terms of DOT, low coverage of this treatment modality was observed in the PEH [10, 24], and it was lower than that in the general population [28], despite evidence of its positive impact on TB treatment success, especially in specific populations, such as the PEH [31, 63, 64]. DOT, along with education, brief messaging, counseling, cash payments and reminders, may improve adherence to TB treatment [65]. In addition, the use of new follow-up technologies, such as video directly observed therapy (VDOT), should be considered, as it could improve coverage [27] and has demonstrated effectiveness in treatment success within the general population. Nevertheless, VDOT faces multifactorial barriers to access for PEH that hinder the feasibility of implementation in this group, a topic that warrants further investigation [66].

Interventions such as DOT and VDOT may be criticized because of their asymmetric biomedical construct. Efforts should be made to shift toward a patient-centered care approach as a cornerstone of TB control. One potential pathway for this transition is the framework developed by Myburgh and collaborators [67], which emphasizes education and psychosocial as well socioeconomic support. This approach aims to achieve, first, the primary goals that patients feel recognized and cared for as persons, empowered to successfully manage their treatment. Additionally, it is crucial to actively promote participation in health decisions and foster relationships of trust with healthcare providers. Interventions must be sensitive to people’s gender, age, socioeconomic status, and health concerns and conditions to obtain instrumental outcomes, such as improved adherence, successful treatment outcomes, and a reduced risk of LFU.

All of the aforementioned elements are important for controlling TB among PEH; however, they represent only some aspects of a multifaceted approach. It should be understood that health is a dynamic and social concept, where social determination of health “is a mode of becoming that produces health processes, within social living that generates the complex dialectic conditioning of health and its corresponding critical processes” with protective and destructive expressions [37]. With this in mind, current models of production and social reproduction, alongside the economic, social, cultural, and political relations that interconnect them, significantly influence individuals’ lifestyles and health profiles [68]. Recognizing that structural factors serve to facilitate and perpetuate health inequalities allows us to redirect the focus of solutions toward removing the structural causes of health inequalities and not holding individuals’ responsibility [69].

### Limitations

This scoping review has several limitations. Although the search took place in six major databases, it is possible that articles of interest to the topic were in other databases. Another limiting aspect was the time frame, leading to the exclusion of good-quality studies published before 2015. The exclusion of qualitative studies was another limitation; however, this was addressed by incorporating qualitative insights into the discussion to capture the experience of the individuals.

Nonetheless, this review synthesizes current evidence and advances knowledge by emphasizing the need for a comprehensive care approach focused on the rights of people. This approach is crucial for the successful treatment of TB in PEH, avoiding solely biomedical focus on the disease, which could perpetuate unfavorable outcomes in this population group.

## Conclusions

The summary of the evidence reaffirms that PEH have lowers rates of TB treatment success and, compared with other population groups, is in the most unfavorable scenario. No study in this review has demonstrated that the ambitious target of a 90% treatment success rate proposed by the WHO for 2025 has been achieved. The proportions of TB treatment outcomes among PEH were variable. The rates of treatment success outcomes ranged from less than 30% to 89.7%. LFU was the most frequently reported negative outcome, followed by “failed” and "not evaluated" treatment outcomes.

Living on the streets, in addition to representing various obstacles for people in this situation, hinders health care access and the cure of a treatable and curable disease such as TB. It is essential to address this reality with a focus on the social determination of health, understanding health as the result of a series of dynamics that permeate people’s actions and depend on the social class and organizational structures of the society in which they live. Multisectoral interventions are necessary to combat poverty and health inequality, which are fundamental factors affecting people’s susceptibility to and management of illness and disease. Sustainable change cannot be achieved without addressing the social inequities that determine health inequalities.

To meet the SDGs and WHO goals and eliminate TB as an epidemic, a comprehensive and collaborative patient-centered care approach is needed. This includes investment in health, social care and education; bringing health services closer to and adapting them for PEH; creating effective links between providers and users; and implementing educational actions to promote, prevent, diagnose, treat TB, and to address stigma and prejudice toward people with TB.

## Supplementary Information

Supplementary Material 1.

## Data Availability

The information database can be consulted on the Open Science Framework at: 10.17605/OSF.IO/PFGE5.

## Abbreviations

TB: Tuberculosis
WHO: World Health Organization
SDGs: Sustainable Development Goals
LFU: Lost to Follow-Up
PEH: People Experiencing Homelessness
PRISMA-ScR: Preferred Reporting Items for Systematic reviews and Meta-Analyses extension for Scoping Reviews
OSF: Open Science Framework
PCC: Population, Concept and Context
MeSH: Medical Subject Headings
DeCS: *Descritores em Ciências da Saúde*
Medline: Medical Literature Analysis and Retrieval System Online
WOS: Web of Science
LILACS: Latin American and Caribbean Health Sciences Literature
CINAHL: Cumulative Index to Nursing and Allied Health Literature
Embase: Excerpa Medica DataBASE
LTBI: Latent Tuberculosis Infection
HR: Hazard Ratio
CI: Incidence Coefficient
OR: Odds Ratio
RR: Risk Ratio
SINAN: *Sistema de Informação de Agravos de Notificação*
RRS: Residential Respite Service
PDL: Population Deprived of Liberty
SciELO: Scientific Electronic Library Online
HIV: Human Immunodeficiency Vírus
AIDS: Acquired Immunodeficiency Syndrome
DOT: Directly Observed Therapy
VDOT: Video Directly Observed Therapy
COVID-19: Coronavirus Disease 2019
