# Supplementary material for: Evidence of tuberculosis treatment outcomes among people experiencing homelessness: a scoping review
Source: BMC Health Serv Res. 2025 Apr 2;25:497. doi: 10.1186/s12913-025-12230-w (PMC11967121; doi:10.1186/s12913-025-12230-w)
Supplement: Supplementary file 1 — Supplementary Material 1. [file 12913_2025_12230_MOESM1_ESM.docx]

**Supplementary material**

**Supplementary material 1** Vocabulary used for the bibliographic search according to the PCC strategy

| **PCC** | **MeSH/DeCS and synonyms** | | |
| --- | --- | --- | --- |
|  | **English** | **Portuguese** | **Spanish** |
| P | Tuberculosis  Infection, Mycobacterium tuberculosis  Infections, Mycobacterium tuberculosis  Koch Disease  Koch's Disease  Kochs Disease  Mycobacterium tuberculosis Infection  Mycobacterium tuberculosis Infections  Tuberculoses | Tuberculose  Mycobacterium tuberculosis  Infecção por Mycobacterium tuberculosis  TB  Pneumologia Sanitária | Tuberculosis  Infección por Mycobacterium tuberculosis  TB |
| C | Treatment Outcome  Clinical Effectiveness  Clinical Efficacy  Effectiveness, Clinical  Effectiveness, Treatment  Efficacy, Clinical  Efficacy, Treatment  Outcome, Patient-Relevant  Outcome, Rehabilitation  Outcome, Treatment  Outcomes, Patient-Relevant  Patient Relevant Outcome  Patient-Relevant Outcome  Patient-Relevant Outcomes  Rehabilitation Outcome  Treatment Effectiveness  Treatment Efficacy  Treatment completion  Treatment Adherence and Compliance | Resultado do tratamento  Efetividade Clínica  Efetividade de Tratamento  Efetividade do Tratamento  Eficácia Clínica  Eficácia de Tratamento  Eficácia do Tratamento  Resultado Relevante ao Paciente  Resultado da Reabilitação  Resultado de Reabilitação  Resultado de Tratamento  Resultados Intermediários de Saúde  Resultados da Promoção de Saúde  Resultados de Intervenções em Saúde  Resultados de Saúde  Conclusão do tratamento  Cooperação e Adesão ao Tratamento | Resultado del Tratamiento  Efectividad Clínica  Efectividad del Tratamiento  Eficacia Clínica  Eficacia del Tratamiento  Rehabilitación Externa  Resultado Relevante al Paciente  Resultado Relevante para el Paciente  Resultado de la Rehabilitación  Resultados Intermedios de Salud  Resultados de Intervenciones en Salud  Resultados de Salud  Resultados de la Promoción de la Salud  Finalización do tratamento  Cumplimiento y Adherencia al Tratamiento |
| C | Ill-Housed Persons  Ill-Housed Person  Ill Housed Persons  Person, Ill-Housed  Persons, Ill-Housed  Shelterless Persons  Person, Shelterless  Persons, Shelterless  Shelterless Person  Homeless Persons  Homeless Person  Person, Homeless  Persons, Homeless  Unhoused Persons  Persons, Unhoused  Person, Unhoused  Unhoused Person  Street People  People, Street  Homelessness  Homeless Shelters  Homeless Shelter  Shelter, Homeless  Shelters, Homeless | Pessoas Mal Alojadas  Falta de Habitação  Falta de Moradia  Morador de Rua  Moradores de Rua  Pessoas em Situação de Rua  Pessoas sem Lar  Pessoas sem-Teto  População em Situação de Rua  Sem-Teto  Pessoa em situação de sem abrigo | Personas con Mala Vivienda  Falta de Hogar  Falta de Vivienda  Gente sin Techo  Personas en Situación de Calle  Personas sin Domicilio Bien Establecido  Personas sin Domicilio Fijo  Personas sin Domicilio Permanente  Personas sin Hogar  Personas sin Refugio  Personas sin Techo  Sin Techo  Sintecho |

**Supplementary material 2** Search strategy by database and vocabulary

| **Database** | **Search strategy** |
| --- | --- |
| Medline  (MeSH) | ("Tuberculos*" OR "Infection, Mycobacterium tuberculosis" OR "Infections, Mycobacterium tuberculosis" OR "Koch Disease" OR "Koch's Disease" OR "Mycobacterium tuberculosis Infection" OR "Mycobacterium tuberculosis Infections" OR "Mycobacterium tuberculosis" OR "TB" OR "Pneumologia Sanitária") AND ("Treatment Outcome" OR "Clinical Effectiveness" OR "Clinical Efficacy" OR "Effectiveness, Clinical" OR "Effectiveness, Treatment" OR "Efficacy, Clinical" OR "Efficacy, Treatment" OR "Outcome, Patient-Relevant" OR "Outcome, Rehabilitation" OR "Outcome, Treatment" OR "Patient Relevant Outcome" OR "Patient-Relevant Outcome" OR "Patient-Relevant Outcomes" OR "Rehabilitation Outcome" OR "Treatment Effectiveness" OR "Treatment Efficacy" OR "Treatment completion" OR "Treatment Adherence and Compliance" OR "Resultado do tratamento" OR "Efetividade do Tratamento" OR "Eficácia Clínica" OR "Resultado de Tratamento" OR "Resultado del Tratamiento" OR "Efectividad Clínica" OR "Efectividad del Tratamiento" OR "Eficacia Clínica" OR "Eficacia del Tratamiento" OR "Cumplimiento y Adherencia al Tratamiento") AND ("Ill-Housed Perso*" OR "Ill Housed Persons" OR "Shelterless Persons" OR "Homeless Perso*" OR "Perso*, Homeless" OR "Unhoused Persons" OR "Unhoused Person" OR "Street People" OR "People, Street" OR "Homeless*" OR "Homeless Shelte*" OR "Shelte*, Homeless" OR "Falta de Vivienda" OR "Personas sin Hogar" OR "Sin Techo" OR "Sintecho") |
| WOS  (MeSH) | ((ALL=("Tuberculos*" OR "Infection, Mycobacterium tuberculosis" OR "Infections, Mycobacterium tuberculosis" OR "Koch Disease" OR "Koch's Disease" OR "Kochs Disease" OR "Mycobacterium tuberculosis Infection" OR "Mycobacterium tuberculosis Infections" OR "Mycobacterium tuberculosis" OR "TB")) AND ALL=("Treatment Outcome" OR "Clinical Effectiveness" OR "Clinical Efficacy" OR "Effectiveness, Clinical" OR "Effectiveness, Treatment" OR "Efficacy, Clinical" OR "Efficacy, Treatment" OR "Outcome, Patient-Relevant" OR "Outcome, Rehabilitation" OR "Outcome, Treatment" OR "Outcomes, Patient-Relevant" OR "Patient Relevant Outcome" OR "Patient-Relevant Outcome" OR "Patient-Relevant Outcomes" OR "Rehabilitation Outcome" OR "Treatment Effectiveness" OR "Treatment Efficacy" OR "Treatment completion" OR "Treatment Adherence and Compliance")) AND ALL=("Ill-Housed Perso*" OR "Ill Housed Persons" OR "Perso*, Ill-Housed" OR "Shelterless Persons" OR "Perso*, Shelterless" OR "Shelterless Person" OR "Homeless Perso*" OR "Perso*, Homeless" OR "Unhoused Persons" OR "Perso*, Unhoused" OR "Unhoused Person" OR "Street People" OR "People, Street" OR "Homeless*" OR "Homeless Shelte*" OR "Shelte*, Homeless" OR "Pessoas Mal Alojadas" OR "Morado* de Rua" OR "Pessoas em Situação de Rua" OR "Pessoas sem Lar" OR "Pessoas sem-Teto" OR "População em Situação de Rua" OR "Sem-Teto" OR "Pessoas em Situação de sem abrigo" OR "Personas en Situación de Calle") |
| Scopus  (MeSH) | ("Tuberculos*" OR "Infection, Mycobacterium tuberculosis" OR "Infections, Mycobacterium tuberculosis" OR "Koch Disease" OR "Koch's Disease" OR "Kochs Disease" OR "Mycobacterium tuberculosis Infection" OR "Mycobacterium tuberculosis Infections" OR "Mycobacterium tuberculosis" OR "Infecção por Mycobacterium tuberculosis" OR "TB" OR "Pneumologia Sanitária" OR "Infección por Mycobacterium tuberculosis") AND ("Treatment Outcome" OR "Clinical Effectiveness" OR "Clinical Efficacy" OR "Effectiveness, Clinical" OR "Effectiveness, Treatment" OR "Efficacy, Clinical" OR "Efficacy, Treatment" OR "Outcome, Patient-Relevant" OR "Outcome, Rehabilitation" OR "Outcome, Treatment" OR "Outcomes, Patient-Relevant" OR "Patient Relevant Outcome" OR "Patient-Relevant Outcome" OR "Patient-Relevant Outcomes" OR "Rehabilitation Outcome" OR "Treatment Effectiveness" OR "Treatment Efficacy" OR "Treatment completion" OR "Treatment Adherence and Compliance" OR "Resultado do tratamento" OR "Efetividade Clínica" OR "Efetividade de Tratamento" OR "Efetividade do Tratamento" OR "Eficácia Clínica" OR "Eficácia de Tratamento" OR "Eficácia do Tratamento" OR "Resultado Relevante ao Paciente" OR "Resultado da Reabilitação" OR "Resultado de Reabilitação" OR "Resultado de Tratamento" OR "Resultados Intermediários de Saúde" OR "Resultados da Promoção de Saúde" OR "Resultados de Intervenções em Saúde" OR "Resultados de Saúde" OR "Conclusão do tratamento" OR "Cooperação e Adesão ao Tratamento" OR "Resultado del Tratamiento" OR "Efectividad Clínica" OR "Efectividad del Tratamiento" OR "Eficacia Clínica" OR "Eficacia del Tratamiento" OR "Rehabilitación Externa" OR "Resultado Relevante al Paciente" OR "Resultado Relevante para el Paciente" OR "Resultado de la Rehabilitación" OR "Resultados Intermedios de Salud" OR "Resultados de Intervenciones en Salud" OR "Resultados de Salud" OR "Resultados de la Promoción de la Salud" OR "Finalización do tratamento" OR "Cumplimiento y Adherencia al Tratamiento") AND ("Ill-Housed Perso*" OR "Ill Housed Persons" OR "Perso*, Ill-Housed" OR "Shelterless Persons" OR "Perso*, Shelterless" OR "Shelterless Person" OR "Homeless Perso*" OR "Perso*, Homeless" OR "Unhoused Persons" OR "Perso*, Unhoused" OR "Unhoused Person" OR "Street People" OR "People, Street" OR "Homeless*" OR "Homeless Shelte*" OR "Shelte*, Homeless" OR "Pessoas Mal Alojadas" OR "Falta de Habitação" OR "Falta de Moradia" OR "Morado* de Rua" OR "Pessoas em Situação de Rua" OR "Pessoas sem Lar" OR "Pessoas sem-Teto" OR "População em Situação de Rua" OR "Sem-Teto" OR "Pessoas em Situação de sem abrigo" OR "Personas con Mala Vivienda" OR "Falta de Hogar" OR "Falta de Vivienda" OR "Gente sin Techo" OR "Personas en Situación de Calle" OR "Personas sin Domicilio Bien Establecido" OR "Personas sin Domicilio Fijo" OR "Personas sin Domicilio Permanente" OR "Personas sin Hogar" OR "Personas sin Refugio" OR "Personas sin Techo" OR "Sin Techo" OR "Sintecho") |
| LILACS  (DeCS) | ("Tuberculos*" OR "Infection, Mycobacterium tuberculosis" OR "Infections, Mycobacterium tuberculosis" OR "Koch Disease" OR "Koch's Disease" OR "Kochs Disease" OR "Mycobacterium tuberculosis Infection" OR "Mycobacterium tuberculosis Infections" OR "Mycobacterium tuberculosis" OR "Infecção por Mycobacterium tuberculosis" OR "TB" OR "Pneumologia Sanitária" OR "Infección por Mycobacterium tuberculosis") AND ("Treatment Outcome" OR "Clinical Effectiveness" OR "Clinical Efficacy" OR "Effectiveness, Clinical" OR "Effectiveness, Treatment" OR "Efficacy, Clinical" OR "Efficacy, Treatment" OR "Outcome, Patient-Relevant" OR "Outcome, Rehabilitation" OR "Outcome, Treatment" OR "Outcomes, Patient-Relevant" OR "Patient Relevant Outcome" OR "Patient-Relevant Outcome" OR "Patient-Relevant Outcomes" OR "Rehabilitation Outcome" OR "Treatment Effectiveness" OR "Treatment Efficacy" OR "Treatment completion" OR "Treatment Adherence and Compliance" OR "Resultado do tratamento" OR "Efetividade Clínica" OR "Efetividade de Tratamento" OR "Efetividade do Tratamento" OR "Eficácia Clínica" OR "Eficácia de Tratamento" OR "Eficácia do Tratamento" OR "Resultado Relevante ao Paciente" OR "Resultado da Reabilitação" OR "Resultado de Reabilitação" OR "Resultado de Tratamento" OR "Resultados Intermediários de Saúde" OR "Resultados da Promoção de Saúde" OR "Resultados de Intervenções em Saúde" OR "Resultados de Saúde" OR "Conclusão do tratamento" OR "Cooperação e Adesão ao Tratamento" OR "Resultado del Tratamiento" OR "Efectividad Clínica" OR "Efectividad del Tratamiento" OR "Eficacia Clínica" OR "Eficacia del Tratamiento" OR "Rehabilitación Externa" OR "Resultado Relevante al Paciente" OR "Resultado Relevante para el Paciente" OR "Resultado de la Rehabilitación" OR "Resultados Intermedios de Salud" OR "Resultados de Intervenciones en Salud" OR "Resultados de Salud" OR "Resultados de la Promoción de la Salud" OR "Finalización do tratamento" OR "Cumplimiento y Adherencia al Tratamiento") AND ("Ill-Housed Perso*" OR "Ill Housed Persons" OR "Perso*, Ill-Housed" OR "Shelterless Persons" OR "Perso*, Shelterless" OR "Shelterless Person" OR "Homeless Perso*" OR "Perso*, Homeless" OR "Unhoused Persons" OR "Perso*, Unhoused" OR "Unhoused Person" OR "Street People" OR "People, Street" OR "Homeless*" OR "Homeless Shelte*" OR "Shelte*, Homeless" OR "Pessoas Mal Alojadas" OR "Falta de Habitação" OR "Falta de Moradia" OR "Morado* de Rua" OR "Pessoas em Situação de Rua" OR "Pessoas sem Lar" OR "Pessoas sem-Teto" OR "População em Situação de Rua" OR "Sem-Teto" OR "Pessoas em Situação de sem abrigo" OR "Personas con Mala Vivienda" OR "Falta de Hogar" OR "Falta de Vivienda" OR "Gente sin Techo" OR "Personas en Situación de Calle" OR "Personas sin Domicilio Bien Establecido" OR "Personas sin Domicilio Fijo" OR "Personas sin Domicilio Permanente" OR "Personas sin Hogar" OR "Personas sin Refugio" OR "Personas sin Techo" OR "Sin Techo" OR "Sintecho") |
| CINAHL  (CINAHL Subjects Headings) | (MH "Tuberculosis" OR "Infection, Mycobacterium tuberculosis" OR "Infections, Mycobacterium tuberculosis" OR "Koch Disease" OR "Koch's Disease" OR "Kochs Disease" OR "Mycobacterium tuberculosis Infection" OR "Mycobacterium tuberculosis Infections" OR "Mycobacterium tuberculosis" OR "Infecção por Mycobacterium tuberculosis" OR "TB" OR "Pneumologia Sanitária" OR "Infección por Mycobacterium tuberculosis") AND (MH "Treatment Outcomes" OR "Clinical Effectiveness" OR "Clinical Efficacy" OR "Effectiveness, Clinical" OR "Effectiveness, Treatment" OR "Efficacy, Clinical" OR "Efficacy, Treatment" OR "Outcome, Patient-Relevant" OR "Outcome, Rehabilitation" OR "Outcome, Treatment" OR "Outcomes, Patient-Relevant" OR "Patient Relevant Outcome" OR "Patient-Relevant Outcome" OR "Patient-Relevant Outcomes" OR "Rehabilitation Outcome" OR "Treatment Effectiveness" OR "Treatment Efficacy" OR "Treatment completion" OR "Treatment Adherence and Compliance" OR "Resultado do tratamento" OR "Efetividade Clínica" OR "Efetividade de Tratamento" OR "Efetividade do Tratamento" OR "Eficácia Clínica" OR "Eficácia de Tratamento" OR "Eficácia do Tratamento" OR "Resultado Relevante ao Paciente" OR "Resultado da Reabilitação" OR "Resultado de Reabilitação" OR "Resultado de Tratamento" OR "Resultados Intermediários de Saúde" OR "Resultados da Promoção de Saúde" OR "Resultados de Intervenções em Saúde" OR "Resultados de Saúde" OR "Conclusão do tratamento" OR "Cooperação e Adesão ao Tratamento" OR "Resultado del Tratamiento" OR "Efectividad Clínica" OR "Efectividad del Tratamiento" OR "Eficacia Clínica" OR "Eficacia del Tratamiento" OR "Rehabilitación Externa" OR "Resultado Relevante al Paciente" OR "Resultado Relevante para el Paciente" OR "Resultado de la Rehabilitación" OR "Resultados Intermedios de Salud" OR "Resultados de Intervenciones en Salud" OR "Resultados de Salud" OR "Resultados de la Promoción de la Salud" OR "Finalización do tratamento" OR "Cumplimiento y Adherencia al Tratamiento") AND (MH "Homeless persons" OR "Ill-Housed Perso*" OR "Ill Housed Persons" OR "Perso*, Ill-Housed" OR "Shelterless Persons" OR "Perso*, Shelterless" OR "Shelterless Person" OR "Homeless Perso*" OR "Perso*, Homeless" OR "Unhoused Persons" OR "Perso*, Unhoused" OR "Unhoused Person" OR "Street People" OR "People, Street" OR "Homeless*" OR "Homeless Shelte*" OR "Shelte*, Homeless" OR "Pessoas Mal Alojadas" OR "Falta de Habitação" OR "Falta de Moradia" OR "Morado* de Rua" OR "Pessoas em Situação de Rua" OR "Pessoas sem Lar" OR "Pessoas sem-Teto" OR "População em Situação de Rua" OR "Sem-Teto" OR "Pessoas em Situação de sem abrigo" OR "Personas con Mala Vivienda" OR "Falta de Hogar" OR "Falta de Vivienda" OR "Gente sin Techo" OR "Personas en Situación de Calle" OR "Personas sin Domicilio Bien Establecido" OR "Personas sin Domicilio Fijo" OR "Personas sin Domicilio Permanente" OR "Personas sin Hogar" OR "Personas sin Refugio" OR "Personas sin Techo" OR "Sin Techo" OR "Sintecho") |
| Embase  (Emtree) | ('tuberculosis' OR 'active TB' OR 'active tuberculosis' OR 'case of TB' OR 'cases of TB' OR 'chronic tuberculosis' OR 'infection by M. tuberculosis' OR 'infection by Mycobacterium tuberculosis' OR 'infection due to M. tuberculosis' OR 'infection due to Mycobacterium tuberculosis' OR 'infection of M. tuberculosis' OR 'infection of Mycobacterium tuberculosis' OR 'Koch`s disease' OR 'M. tuberculosis infection' OR 'minimal tuberculosis' OR 'minimum tuberculosis' OR 'Mycobacterium tuberculosis infection' OR 'TB (tuberculosis)' OR 'TB case' OR 'TB cases' OR 'TB disease' OR 'TB infection' OR 'tuberculous infection' OR 'tuberculous lesion' OR 'tuberculosis' OR 'Infecção por Mycobacterium tuberculosis' OR 'TB' OR 'Pneumologia Sanitária' OR 'Infección por Mycobacterium tuberculosis') AND ('Treatment outcome' OR 'health care outcome and process assessment' OR 'healthcare outcome and process assessment' OR 'medical futility' OR 'outcome and process assessment (health care)' OR 'outcome and process assessment, health care' OR 'outcome management' OR 'patient outcome' OR 'therapeutic outcome' OR 'therapy outcome' OR 'Treatment Adherence and Compliance' OR 'Resultado do tratamento' OR 'Efetividade Clínica' OR 'Efetividade de Tratamento' OR 'Efetividade do Tratamento' OR 'Eficácia Clínica' OR 'Eficácia de Tratamento' OR 'Eficácia do Tratamento' OR 'Resultado Relevante ao Paciente' OR 'Resultado da Reabilitação' OR 'Resultado de Reabilitação' OR 'Resultado de Tratamento' OR 'Resultados Intermediários de Saúde' OR 'Resultados da Promoção de Saúde' OR 'Resultados de Intervenções em Saúde' OR 'Resultados de Saúde' OR 'Conclusão do tratamento' OR 'Cooperação e Adesão ao Tratamento' OR 'Resultado del Tratamiento' OR 'Efectividad Clínica' OR 'Efectividad del Tratamiento' OR 'Eficacia Clínica' OR 'Eficacia del Tratamiento' OR 'Rehabilitación Externa' OR 'Resultado Relevante al Paciente' OR 'Resultado Relevante para el Paciente' OR 'Resultado de la Rehabilitación' OR 'Resultados Intermedios de Salud' OR 'Resultados de Intervenciones en Salud' OR 'Resultados de Salud' OR 'Resultados de la Promoción de la Salud' OR 'Finalización do tratamento' OR 'Cumplimiento y Adherencia al Tratamiento') AND ('homelessness' OR 'homeless status' OR 'living on the streets' OR 'homeless households' OR 'homeless people' OR 'homeless persons' OR 'homeless population' OR 'ill-housed people' OR 'ill-housed persons' OR 'people living on the streets' OR 'shelterless persons' OR 'street people' OR 'unhoused persons' OR 'vagabond' OR 'vagrant people' OR 'vagrant person' OR 'homeless person' OR 'Pessoas Mal Alojadas' OR 'Falta de Habitação' OR 'Falta de Moradia' OR 'Morado* de Rua' OR 'Pessoas em Situação de Rua' OR 'Pessoas sem Lar' OR 'Pessoas sem-Teto' OR 'População em Situação de Rua' OR 'Sem-Teto' OR 'Pessoas em Situação de sem abrigo' OR 'Personas con Mala Vivienda' OR 'Falta de Hogar' OR 'Falta de Vivienda' OR 'Gente sin Techo' OR 'Personas en Situación de Calle' OR 'Personas sin Domicilio Bien Establecido' OR 'Personas sin Domicilio Fijo' OR 'Personas sin Domicilio Permanente' OR 'Personas sin Hogar' OR 'Personas sin Refugio' OR 'Personas sin Techo' OR 'Sin Techo' OR 'Sintecho') |
